# Low BMI-1 expression is associated with an activated BMI-1-driven signature, vascular invasion, and hormone receptor loss in endometrial carcinoma

**DOI:** 10.1038/sj.bjc.6604360

**Published:** 2008-05-13

**Authors:** I B Engelsen, M Mannelqvist, I M Stefansson, S L Carter, R Beroukhim, A M Øyan, A P Otte, K H Kalland, L A Akslen, H B Salvesen

**Affiliations:** 1 Department of Obstetrics and Gynecology, Haukeland University Hospital, Bergen 5021, Norway; 2 The Gade Institute, University of Bergen, Postboks 7800, Bergen 5020, Norway; 3 Institute of Clinical Medicine, University of Bergen, Postboks 7800, Bergen 5020, Norway; 4 Department of Pathology, Haukeland University Hospital, Bergen 5021, Norway; 5 The Harvard MIT Division of Health Sciences and Technology, Boston, MA, USA; 6 Broad Institute of Harvard and MIT, Boston, MA, USA; 7 Department of Medical Oncology, Dana-Farber Cancer Institute, Boston, MA, USA; 8 Department of Microbiology and Immunology, Haukeland University Hospital, Bergen 5021, Norway; 9 Department of Biochemistry, Swammerdam Institute for Life Sciences, University of Amsterdam, Amsterdam, The Netherlands

**Keywords:** BMI-1, endometrial carcinoma, immunohistochemistry, mRNA, gene signature, hormone receptors

## Abstract

We studied the expression of polycomb group (PcG) protein BMI-1 in a large population-based patient series of endometrial carcinomas in relation to clinical and molecular phenotype. Also, 57 fresh frozen endometrial carcinomas were studied for the relationship between BMI-1 protein expression, BMI-1 mRNA level, and activation of an 11-gene signature reported to represent a BMI-1-driven pathway. BMI-1 protein expression was significantly weaker in tumours with vascular invasion (*P*<0.0001), deep myometrial infiltration (*P*=0.004), and loss of oestrogen receptor (ER) (*P*<0.0001) and progesterone receptors (PR) (*P*=0.03). Low BMI-1 protein expression was highly associated with low BMI-1 mRNA expression (*P*=0.002), and similarly low BMI-1 mRNA expression correlated significantly with vascular invasion, ER and PR loss, and histologic grade 3. In contrast, activation of the reported 11-gene signature, supposed to represent a BMI-1-driven pathway, correlated with low mRNA expression of BMI-1 (*P*<0.001), hormone receptor loss, presence of vascular invasion, and poor prognosis. We conclude that BMI-1 protein and mRNA expression are significantly correlated and that BMI-1 expression is inversely associated with activation of the 11-gene signature. Loss of BMI-1 seems to be associated with an aggressive phenotype in endometrial carcinomas.

Endometrial cancer is one of the most common malignant tumours of the female genital tract and its incidence is increasing in developed countries (WHO, 2003). The molecular pathogenesis of endometrial carcinoma remains poorly understood, but it is widely believed that a common feature of human cancer cells is their ability to bypass control mechanisms restricting proliferation, apoptosis, angiogenesis, invasion, and metastases (Hanahan and Weinberg, 2000; Lax, 2004; Ryan *et al*, 2005; Prat *et al*, 2007). Over the years, several reports on endometrial cancer and prognostic factors have been published, and the impact of age, histologic type and grade, Federation of Gynecology and Obstetrics (FIGO) stage, ploidy, and hormone receptor status is well established (Amant *et al*, 2005).

BMI-1 is a member of the polycomb group (PcG) of genes that are important transcriptional regulators by the formation of PcG bodies and chromatin remodelling (Shao *et al*, 1999). BMI-1 acts through epigenetic silencing by organising the chromatin into an inaccessible structure that cannot bind transcription factors, and it may, in fact, also act by repressing cyclin-dependent kinase inhibitors *p16/INK4a* and *p14/ARF* (Jacobs *et al*, 1999; Jacobs and van Lohuizen, 2002; Pasini *et al*, 2004; Liu *et al*, 2006).

Recent studies have promoted BMI-1 as a stem cell marker that regulates self-renewal in haematopoietic and leukaemic stem cells (Lessard and Sauvageau, 2003; Raaphorst, 2003). Elevated expression of human BMI-1 has also been reported in multiple cancer samples by immunohistochemistry or mRNA analysis for oral cancers (Kang *et al*, 2007), lung cancers (Vonlanthen *et al*, 2001; Breuer *et al*, 2004), lymphomas (Dukers *et al*, 2004; Raaphorst *et al*, 2004), and breast cancer (Kim *et al*, 2004; Silva *et al*, 2006) as well as in cancer cell lines (Satijn *et al*., 1997; Satijn and Otte, 1999), although the exact function is not known.

Global gene expression profiling can be used to identify molecular signatures associated with activation of certain oncogenic pathways (Huang *et al*, 2003). Glinsky *et al* (2005) described the prognostic impact of an 11-gene signature displaying a stem cell-resembling expression profile. Their data indicate the presence of a conserved BMI-1-driven pathway, which is similarly engaged in normal stem cells and in a series of highly malignant human cancers arising from a wide range of organs. Steele *et al* (2006) used SEREX technology and proved widespread overexpression of BMI-1 protein in different tumour types. The role of BMI-1 in endometrial carcinomas is unknown.

On the basis of this background, we have investigated the expression of BMI-1 in endometrial carcinomas in relation to histopathologic features, markers of cell-cycle regulation, and hormone receptors, as well as patient outcome. In particular, we wanted to investigate the relationship between BMI-1 protein expression, BMI-1 mRNA levels, and activation of the 11-gene signature reported to represent a BMI-1-driven pathway (Glinsky *et al*, 2005) in relation to the clinical and molecular phenotype.

## MATERIALS AND METHODS

Two independent population-based patient series have been studied for protein expression of BMI-1 and the prognostic relevance of traditional and newly described markers. In a retrospectively collected patient series, all 316 patients diagnosed with endometrial carcinoma in Hordaland County of Western Norway from 1981 to 1990 were studied (patient series 1). Paraffin blocks from the primary tumour were available in 286 cases (96%). In this particular study, 264 cases had sufficient material in the tissue microarray (TMA) blocks for the immunohistochemical study of BMI-1 expression using two different antibodies. Data from previously studied tumour markers, such as vascular invasion, growth pattern, depth of myometrial infiltration, tumour cell proliferation, DNA index, oestrogen receptor (ER), progesterone receptor (PR), HER-2/neu, p53, and p16 expression, were available for comparisons (Salvesen *et al*, 1999, 2000; Stefansson *et al*, 2004b). The patient material and treatment protocol of this series are described in detail elsewhere (Salvesen *et al*, 1999; Engelsen *et al*, 2006).

The follow-up data were collected from the medical records and correspondence with the primary physicians. The median follow-up period for the survivors was 9 years (range 5–15 years). None of the patients were lost due to insufficient follow-up information. Among the 117 patients who died during the follow-up period, 70 died from endometrial carcinoma, whereas 47 died from other causes. The follow-up data were cross-checked with information from the Cancer Registry of Norway, which is matched against the Register of Deaths of Statistics Norway. The last date of follow-up was 30 June 1996.

To further explore the BMI-1 mRNA and protein expression, and the reported 11-gene signature for a BMI-1-driven pathway, fresh endometrial carcinoma tissue was collected prospectively from 57 randomly selected patients from a population-based tissue bank collected in the period 2001–2004 (patient series 2). Clinicopathologic data such as age at the time of diagnosis, International FIGO stage, according to the 1988 criteria, histologic type and histologic grade, vascular invasion, myometrial infiltration, presence of lymph node metastases, and treatment and survival were recorded. Patients were followed from the time of primary surgery until June 2007 or until death. Median follow-up time for survivors was 3.6 years (range 0.8–5.5 years). To elucidate potential selection bias for the prospectively collected patient series, the traditional clinicopathologic variables were compared to the corresponding data for the initial complete population-based series from 1981 to 1990, and no significant differences in patient characteristics were detected (Supplementary Table 1).

### Tissue microarray

Tumour specimens from both patient series studied were mounted in TMA as previously described and validated in several studies (Hoos *et al*, 2001; Straume and Akslen, 2002; Stefansson *et al*, 2004a). Tissue microarray construction was made by identifying the area of highest tumour grade on H&E-stained slides, followed by punching out three tissue cylinders from the selected areas of the donor block and then mounted into a recipient paraffin block using a custom-made precision instrument (Beecher Instruments, Silver Spring, MD, USA).

### Immunohistochemistry

The immunohistochemical staining was performed on thin TMA sections (5 *μ*m) of paraffin-embedded tissue. The slides were dewaxed with xylene/ethanol before microwave antigen retrieval for 10 min at 750 W and 15 min at 350 W in TE 9 buffer (pH 9). The slides were incubated overnight at 4°C with a monoclonal BMI-1 antibody (clone F6; Upstate, Lake Placid, NY, USA) diluted 1 : 800. Slides incubated with Mouse IgG1 diluted 1 : 800 were used as negative controls. Samples of breast and prostate cancers with known BMI-1 positivity were used as positive controls. The staining was performed using the EnVision-labelled polymer method, with a commercial kit (Dako Cytomation, Copenhagen, Denmark), with 3-amino-9-ethylcarbazole (AEC) peroxidase as substrate before brief counterstaining with Mayer's haematoxylin. The TMA sections were also stained with a well-described non-commercial monoclonal anti-BMI-1 antibody (Breuer *et al*, 2004; Dukers *et al*, 2004). After pretreatment and antigen retrieval as described above, the slides were incubated for 60 min at room temperature with the undiluted monoclonal antibody (6C9) before completion of the staining procedure using the Catalyzed Signal Amplification System (Dako Cytomation) in line with the instructions from the manufacturer.

The arrays were scored blinded for clinical information and results from the other markers studied, and there was good correlation between the two investigated antibodies (Pearson's correlation coefficient 0.64, *P*<0.0001).

### Evaluation of staining

The immunohistochemical staining of BMI-1 showed a predominantly nuclear staining pattern. The staining was recorded using a semiquantitative and subjective grading, considering both the intensity of staining and the proportion of tumour cells showing unequivocal positive reaction. A staining index (SI) was calculated as a product of staining intensity (0–3) and area of positive tumour cell nuclei (1=<10%, 2=10–50%, 3=>50%) (Aas *et al*, 1996; Straume and Akslen, 1997). Evaluation of the cases was carried out blinded for patient characteristics and outcome. In subsequent statistical analyses, the cut-off was based on the median value for the SI, after considering the frequency distribution curve and the size of subgroups.

### Microarray analysis

Surgically removed tumour biopsies were quick frozen and subsequently ground to powder in a mortar under liquid N_2_. Total RNA was extracted according to standard protocols (Invitrogen (Carlsbad, CA, USA) Trizol LS protocol and Qiagen (Hilden, Germany) RNeasy Mini Kit protocol). Assessment of quality and yield of total RNA, extraction of poly(A) RNA, and preparation of Cy-labelled hybridisation targets have been described in detail (Petersen *et al*, 2007). Approximately 500 ng each of endometrial Cy^3^-labelled aminoallyl-cRNA and Stratagene Universal RNA Cy^5^-labelled aminoallyl-cRNA at a specific labelling of about 100 pmol Cy per *μ*g cRNA were combined, fragmented, and hybridised with the Agilent 21 k (batch 1) and 22 k human oligonucleotide microarrays (batch 2) according to the manufacturer's protocol (Agilent Technologies, Santa Clara, CA, USA). After hybridisation at 60°C for 17 h, the stringent wash was done in 50 ml 0.1 × SSC at 35°C for 10 min followed by three quick washes in 0.1 × SSC at room temperature. The oligonucleotide microarrays were scanned and features automatically extracted, recorded, and analysed using the Agilent Microarray Scanner Bundle.

mRNA was extracted from surgically dissected and fresh frozen primary tumours from clinically well-defined endometrial carcinomas. All primary tumour specimens were examined histologically to ensure at least 50% neoplastic tissue, but the majority of the samples showed at least 80% neoplastic tissue.

### Batch adjustment

As the samples were hybridised in two rounds on two different array print batches and initial clustering of the samples indicated batch effects (data not shown), the data were adjusted to minimise this effect. A representative subset of samples with similar biology, which was based on a selection of external variables, was selected from each batch and formed the basis for batch adjustment. Then for each gene, the expression levels of each sample batch was adjusted such that the mean expression level of the representative subset was set to 0 (Lapointe *et al*, 2004).

### Real-time quantitative PCR estimation of mRNA

mRNA expression for BMI-1 and selected markers, such as ER*α*, PR, p16, p53, and HER-2/neu, were validated by quantitative PCR (qPCR) using the TaqMan® Low Density Array (TLDA) technique (Rostad *et al*, 2007). TaqMan Low Density Arrays are customisable, 384-well microfluidic cards for real-time qPCR (Applied Biosystems, Foster City, CA, USA). TaqMan Low Density Array cards were constructed with two control genes (*GAPDH* and *ACTB* (β-actin), and the selected genes were analysed in duplicate. Single-stranded cDNA for qPCR analysis was synthesised from 2 *μ*g of total RNA using a final concentration of 5 *μ*M random hexamer primers, pd (N)_6_ (GE Healthcare Life Sciences, Uppsala, Sweden) and M-MLV reverse transcriptase according to Ambion instructions (Applied Biosciences, Foster City, CA, USA). Complementary DNA corresponding to 40 ng of endometrial total RNA was mixed with TaqMan Universal buffer and added to each TLDA loading well as recommended (Applied Biosystems). The PCR reaction was run at 50°C for 2 min, 95°C for 10 min, and 40 cycles at 95°C for 15 s and 60°C for 60 s. The SDS 2.2 software was used for data analysis.

### Activation of a gene signature for BMI-1-driven pathway

In our microarray data set, 9 out of 11 genes in the BMI-1-associated signature reported by Glinsky *et al* (2005) were identified and used to see if the BMI-1 signature activation was related to the phenotype of endometrial carcinomas. The BMI-1 activation signature contains eight upregulated genes, *GBX2*, *MKI67*, *CCNB1*, *BUB1*, *KNTC2*, *USP22*, *HCFC1*, and *RNF2*, and three downregulated genes, *ANK3*, *FGFR2*, and *CES1*, of which all but *KNTC2* and *USP22* were available in the present data set (Figure 4). Expression data were normalised by subtracting the mean expression of each gene and then dividing by the s.d. The signature was then evaluated by computing the sum of normalised expression values for the upregulated genes minus the sum of the downregulated genes.

### Validation of gene signature for BMI-1-driven pathway in external data set

Publicly available gene expression data corresponding to tumours resected from uterine tissue were obtained from the TGEN expression profiling for oncology (http://expo.intgen.org/geo/home.do). GEO accession numbers for these tumours are listed in Supplementary Table 2. One hundred and four of the uterine tumours had information regarding histologic subtype available, 91 endometrioid and 13 non-endometrioid tumours, whereas 68 tumours had information regarding histologic grade available, 39 grade 1/2 tumours, and 29 grade 3 tumours. Survival data were not available for these patients. All 11 genes in the BMI-1-associated signature reported by Glinsky *et al* (2005) were identified in this data set and were used to see if our finding that BMI-1 signature activation was related to the phenotype of endometrial carcinomas could be validated in external data.

### Statistics

Analyses were performed by the statistical software package SPSS 14.0. Associations between different variables were assessed by the Mann–Whitney test, Pearson's *χ*^2^ test, and linear regression. Univariate analyses of time to recurrence (recurrence free survival) and death due to endometrial carcinoma (disease-specific survival) were performed using the Kaplan–Meier method. Differences in survival between categories were estimated by the log-rank (Mantel Cox) test. The cut-off was based on the median value for the SI and mRNA expression levels, after considering the frequency distribution curve and the size of the subgroups. The tumour samples were analysed for expression level for 9 out of 11 genes in the BMI-1 activation signature available in the data set using the J-Express tool (Dysvik and Jonassen, 2001). Before clustering, the expression profiles of each gene were centred by subtracting the mean over all samples. The genes were subjected to hierarchical clustering using average linkage (WPGMA) and Pearson's correlation as similarity measures. The resulting unsupervised clustering reveals two main clusters of samples, which are referred to as cluster 1 and cluster 2 (Figure 4).

## RESULTS

### Immunohistochemical staining for BMI-1

One hundred and forty-four (55%) of 264 patients in the retrospective population-based patient series showed strong nuclear staining in >50% of the tumour cells. BMI-1 expression (Clone F6) was significantly lower in tumours with the presence of vascular invasion (*P*<0.0001) and deep myometrial infiltration (*P*=0.004) (Table 1). Low BMI-1 expression was also significantly associated with the loss of immunohistochemical staining for ER (*P*<0.0001) and PR (*P*=0.03) (Table 1) and for tumours with diffusely infiltrative growth pattern when compared to those with pushing border (*P*=0.01) (Stefansson *et al*, 2006). When analysing the non-commercial BMI-1 antibody (Breuer *et al*, 2004; Dukers *et al*, 2004), similar results were obtained (data not shown). For the other clinicopathologic variables (FIGO stage, histologic subtype, and histologic grade), we found no significant associations.

### mRNA estimation of BMI-1 by qPCR

**Figure 1:**
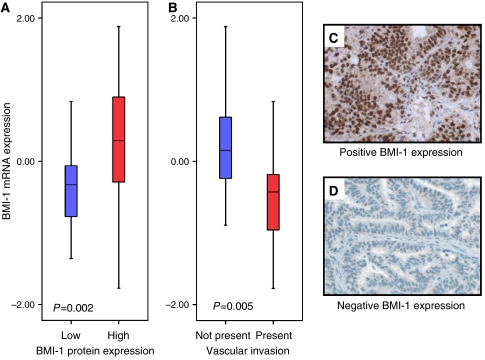
Box plot illustrating correlation between BMI-1 protein expression and mRNA BMI-1 expression. Among samples with low BMI-1 protein expression, there is a highly significantly lower level of BMI-1 mRNA expression (**A**). Cases with vascular invasion show significantly lower level of BMI-1 mRNA expression (**B**). Low BMI-1 expression was seen in 25 (44%) samples compared with high expression in 32 (56%) samples. Examples of high immunohistochemical BMI-1 expression are illustrated in panel C and low expression in panel D.

**Figure 2:**
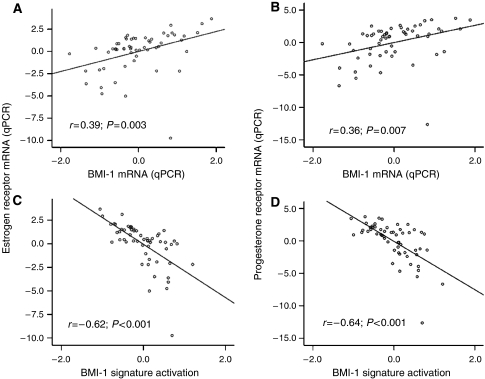
Scatter plot of mRNA expression by qPCR for oestrogen receptor (**A**) and progesterone receptor (**B**) in relation to BMI-1 mRNA expression (qPCR) and mRNA expression of the 11-gene BMI-1 signature (**C** and **D**). A regression line is drawn to illustrate the relationship.

mRNA expression of BMI-1 estimated by microarray and qPCR was significantly correlated (*r*=0.56 (Pearson's); *P*<0.001, Supplementary Figure 1). Low immunohistochemical staining for BMI-1 was significantly associated with low mRNA BMI-1 expression (*P*=0.002, Figure 1A). We found a highly significant association between the presence of vascular invasion and low BMI-1 mRNA expression (*P*=0.005, Figure 1B, Table 2). Low mRNA expression was also associated with high histologic grade (*P*=0.02), whereas the association with non-endometrioid histologic subtype was of borderline significance (*P*=0.07). There was no association with FIGO stage or myometrial infiltration (Table 2). Owing to the identified correlations between BMI-1 expression and steroid hormones in the immunohistochemical studies, we also investigated the correlation between mRNA expression for BMI-1 and ER*α*/PR. A highly significant correlation between BMI-1 and ER*α* (*r*=0.39; *P*=0.003) and PR (*r*=0.36; *P*=0.007) was confirmed (Figure 2A and B).

### Activation of a gene signature for BMI-1-driven pathway

**Figure 3:**
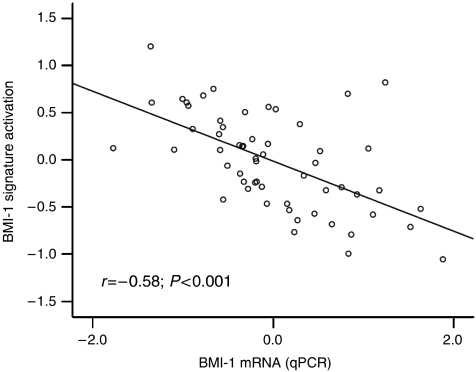
Scatter plot of BMI-1 mRNA expression by qPCR and the level of the reported 11-gene BMI-1 signature. A regression line is drawn to illustrate the relationship.

**Figure 4:**
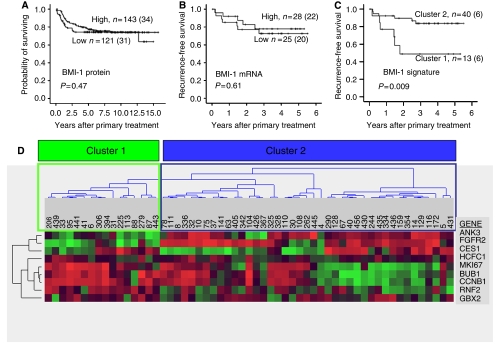
Expression of (**A**) BMI-1 protein, (**B**) BMI-1 mRNA, and (**C**) cluster for BMI signature activation related to survival. Survival curves estimated according to the Kaplan–Meier method with death due to endometrial carcinoma as end point in panel A and time to recurrent disease as end point in panels B and C. Nine out of 11 genes in the BMI-1 signature were identified in the present data set. Unsupervised hierarchical clustering (WPGMA; Pearson's correlation) gives a cluster formation where tumours in cluster 1 have significantly lower recurrence-free survival as compared with tumours in cluster 2 (*P*=0.009, **C** and **D**).

The endometrial carcinomas in the present data set were then investigated for activation of a BMI-1-driven signature reported by Glinsky *et al* (2005). Nine out of 11 genes were available in our microarray data sets, and the activation level of this signature was inversely correlated with mRNA expression of BMI-1 (*r*=−0.58; *P*<0.001, Figure 3), ER*α* (*r*=−0.62; *P*<0.001), and PR (*r*=−0.64; *P*<0.001) (Figure 2C and D). BMI-1 activation signature was significantly correlated to non-endometrioid histologic subtype (*P*=0.001), high histologic grade (grade 3 *vs* 1/2) (*P*=0.001), vascular invasion (*P*=0.04), and poor patient prognosis (*P*=0.009) (Table 3 and Figure 4C and D). When correlating histologic subtype and grade to activation of the BMI-1-driven signature in external gene expression data, activation of this signature confirmed it to be significantly correlated to non-endometrioid histologic subtype (*P*=0.02) and high histologic grade (grade 3) (*P*=0.001).

### Survival analyses

We analysed both patient series for a survival impact of BMI-1 expression in univariate survival analysis. Neither of the two antibodies used for immunohistochemical staining nor the mRNA expression was associated with patient survival. In contrast, unsupervised clustering based on 9 out of 11 genes in the BMI-1 signature revealed two distinct clusters, and the cluster with activation of the BMI-1-related signature was highly significantly associated with poor prognosis (*P*=0.009) (Figure 4C).

## DISCUSSION

This is, to the best of our knowledge, the first study on BMI-1 expression in endometrial carcinomas. Further, this is the first study to relate BMI-1 protein expression to BMI-1 mRNA expression and the level of activation for the previously reported BMI-1 signature in the same tumours. High BMI-1 expression, estimated both by immunohistochemical staining and by qPCR for mRNA levels, was seen in a subgroup of endometrial carcinomas. This appears to be in line with other studies showing high BMI-1 expression in a range of malignant tumours, such as oral cancer, lung cancer, Hodgkin's lymphoma, breast cancer, hepatocellular cancer and prostate cancer (Vonlanthen *et al*, 2001; Breuer *et al*, 2004; Dukers *et al*, 2004; Glinsky *et al*, 2005; Shi *et al*, 2005; Steele *et al*, 2006; Kang *et al*, 2007; Silva *et al*, 2007). Also, the immunohistochemical expression pattern in endometrial tumours was concordant with the mRNA levels for BMI-1, indicating the relevance of BMI-1 protein in this tumour type.

The importance of BMI-1 for tumour progression in endometrial carcinomas is unknown. We find that the loss of BMI-1 protein and loss of mRNA expression are highly significantly correlated. The correlation between low expression of BMI-1 protein and histological grade 3 and non-endometrioid subtype is weaker and not significant as compared with the significant or borderline significant correlation for low mRNA expression and these two factors. There is, however, a pattern in the same direction for BMI-1 protein expression. BMI-1 mRNA expression is estimated as a continuous variable, and this may increase the ability to detect differences. We find that the low immunohistochemical expression and mRNA level of BMI-1 was significantly correlated to the presence of vascular invasion, suggesting a more aggressive phenotype. An association between BMI-1 and vascular involvement has not been previously reported. Further, in our studies, low BMI-1 protein and mRNA expression were significantly associated with low expression of the steroid receptors ER and PR. This has previously been reported for breast cancer in some studies (Kim *et al*, 2004; Arnes *et al*, 2008), but not in others (Silva *et al*, 2007). Still, this could indicate a potential link between BMI-1 and hormone receptor status. Paradoxically, high expression of BMI-1 was significantly associated with positive ER status and with the presence of lymph node metastasis in one study (Kim *et al*, 2004), indicating a diverse role of BMI-1. Overexpression of BMI-1 has shown a prognostic impact on survival only in some studies but not all (Silva *et al*, 2006; Song *et al*, 2006).

In our study, BMI-1 did not show any correlation with downstream target p16, indicating suppression. The lack of association between BMI-1 and p16 expression has also been reported for breast cancer (Silva *et al*, 2006), lung cancer (Breuer *et al*, 2005), and Hodgkin's lymphoma (Dukers *et al*, 2004), whereas in oral cancer (Kang *et al*, 2007) and nasopharyngeal cancer (Song *et al*, 2006), BMI-1 has been indicated to act through p16 to regulate cellular proliferation. Liu *et al* (2006) recently showed that in cell line studies for different tissues that in both normal and cancer cells, the loss of BMI-1 led to the upregulation of p16, but with no significant effect on the level of telomerase gene expression. This suggests that other BMI-1 cooperative factors may be involved in the BMI-1-dependent cancer-specific growth retardation.

The reported signature by Glinsky *et al* (2005) has been assumed to represent an activated BMI-1-driven pathway, and it has been linked to poor survival for a range of cancers. We found a significant association between the activation of this Glinsky signature and non-endometrioid histologic subtype, histologic grade 3, vascular invasion, and survival in line with what has been reported for other cancer types (Glinsky *et al*, 2005). The correlation with histologic subtype and grade was also validated in external data. Thus, our findings confirm an association between the activated 11-gene signature and an aggressive phenotype for endometrial cancer, and this has not been previously shown for this cancer type.

In contrast, we found that low BMI-1 expression was associated with high-risk factors such as histologic grade 3, vascular invasion, and loss of hormone receptors. It appears to be counter-intuitive that expression levels for BMI-1 by mRNA is inversely correlated to the expression level for the Glinsky signature reported to represent a BMI-1-driven pathway. The finding that low BMI-1 expression is associated with several markers of poor prognosis is fairly consistent both for protein and for mRNA expression. A series of complex signalling pathways may influence the expression level of the Glinsky signature. Still, on the basis of our results, this regulation seems to be more complex than by a direct effect through BMI-1 mRNA expression. The critical factors driving the expression level for this signature are not known and have not previously been studied for endometrial carcinomas. Our findings may suggest that this signature does not represent only a BMI-1-driven pathway for endometrial carcinomas.

Although Glinsky *et al* found low BMI-1 mRNA expression to be associated with less aggressive tumours of prostate cancer, a finding that is supported by others in studies of non-small-cell lung cancer and breast carcinomas (Vonlanthen *et al*, 2001; Raaphorst *et al*, 2003), we found that low BMI-1 expression is associated with markers of a more aggressive phenotype in endometrial cancer, even though no significant prognostic impact of BMI-1 expression is seen.

Glinsky *et al* claim that the signature represents a ‘stem’-like cellular state, required for self-renewal. However, some of the genes, such as *MKI67*, *CCNB1*, and *BUB1*, have also been reported to come up in standard proliferation signatures (Moreno-Bueno *et al*, 2003) and signatures for chromosomal instability (Carter *et al*, 2006), which may also explain the prognostic influence of this reported signature. All of these biologic processes may be closely related, and it may not be possible to separate them, as BMI-1 has also been found to be important for chromatin remodelling (Shao *et al*, 1999) and may affect proliferation by repressing the cyclin-dependent kinase inhibitors *p16/INK4a* and *p14/ARF* (Jacobs *et al*, 1999; Jacobs and van Lohuizen, 2002; Pasini *et al*, 2004; Liu *et al*, 2006).

In conclusion, we demonstrate for the first time that loss of BMI-1 expression is significantly correlated with vascular invasion and loss of hormone receptors in endometrial carcinomas. This could indicate a protective role of the BMI-1 protein in this tumour type. BMI-1 mRNA expression is inversely correlated to the level of activation by the reported BMI-1 activation signature, supposed to represent a BMI-10driven pathway. We then show that activation of this signature is associated with an aggressive phenotype and survival in endometrial carcinomas. Our findings justify further studies to validate the role of BMI-1 in endometrial carcinomas. Also, studies of the possible value of BMI-1 in novel treatment strategies for this disease are needed.

## Figures and Tables

**Table 1:** BMI-1 protein expression in tumours related to clinicopathologic variables and biomarkers in a population-based study of endometrial carcinomas^a^

	**BMI-1 expression^b^**		
	**Low**	**High**	
**Variable**	***n* (%)**	***n* (%)**	***P-*value**
*FIGO stage*			0.39
I/II	118 (56)	94 (44)	
III/IV	25 (49)	26 (51)	
			
*Histologic subtype*			0.43
Endometrioid	127 (53)	111 (47)	
Non-endometrioid	16 (62)	10 (38)	
			
*Histologic grade (FIGO)*			0.38
Grades 1–2	112 (53)	100 (47)	
Grade 3	31 (60)	21 (40)	
			
*Vascular invasion*			<0.0001
Not present	74 (44)	93 (56)	
Present^c^	69 (71)	28 (29)	
			
*Myometrial infiltration*			0.004
<50%	56 (49)	58 (51)	
⩾50%	50 (70)	21 (30)	
			
*ER*			0.001
Low expression	44 (77)	13 (23)	
High expression	85 (51)	82 (49)	
			
*PR*			0.03
Low expression	46 (69)	21 (31)	
High expression	84 (53)	74 (47)	

ER=estrogen receptor; FIGO=Federation of Gynecology and Obstetrics; PR=progesterone receptor.

a Two hundred and sixty-four cases with evaluable staining for BMI-1 (Upstate), missing data for FIGO stage in 1 case, depth of myometrial infiltration in 79 cases, for ER in 40 cases, and for PR in 39 cases.

b Median used as cut-off value.

c Vascular invasion of tumour cells detected in ⩾2 vessels.

**Table 2:** BMI-1 mRNA expression estimated by qPCR in 57 prospectively collected, fresh-frozen endometrial cancers in relation to clinicopathologic variables

**Variable**	***n***	**Median**	***P*-value**
*FIGO stage*			
I/II	48	−0.09	0.19
III/IV	9	−0.33	
			
*Histologic type*			
Endometrioid	51	−0.11	0.07
Non-endometrioid	6	−0.65	
			
*Histologic grade*			
Grade 1/2	44	−0.07	0.02
Grade 3	13	−0.66	
			
*Vascular invasion*			
Not present	35	0.15	0.005
Present^a^	22	−0.43	
			
*Myometrial infiltration*			
<50%	30	−0.09	0.76
⩾50%	27	−0.20	

FIGO=Federation of Gynecology and Obstetrics; qPCR=quantitative PCR.

a Vascular invasion detected in ⩾2 vessels.

**Table 3:** BMI-1 11-gene signature activation based on mRNA expression by microarray data in 57 prospectively collected fresh-frozen endometrial cancer tumours in relation to clinicopathologic variables

**Variable**	***n***	**Median**	***P*-value**
*FIGO stage*			
I/II	48	−0.04	0.1
III/IV	9	0.27	
			
*Histologic type*			
Endometrioid	51	−0.06	0.01
Non-endometrioid	6	0.58	
			
*Histologic grade*			
Grade 1/2	44	−0.20	0.001
Grade 3	13	0.56	
			
*Vascular invasion*			
Not present	35	−0.23	0.04
Present^a^	22	−0.13	
			
*Myometrial infiltration*			
<50%	30	−0.02	0.28
⩾50%	27	−0.14	

FIGO=Federation of Gynecology and Obstetrics.

a Vascular invasion detected in ⩾2 vessels.
