# Supplementary material for: Low BMI-1 expression is associated with an activated BMI-1-driven signature, vascular invasion, and hormone receptor loss in endometrial carcinoma
Source: Br J Cancer. 2008 May 13;98(10):1662–9. doi: 10.1038/sj.bjc.6604360 (PMC2391115; doi:10.1038/sj.bjc.6604360)
Supplement: Supplementary Table 2 [file 6604360x2.doc]

**Supplementary Table 2:**
